# Hybrid emergency care at the home for patients – A multiple case study

**DOI:** 10.1186/s12873-024-01087-7

**Published:** 2024-09-16

**Authors:** Åsa Falchenberg, Ulf Andersson, Gabriella Norberg Boysen, Henrik Andersson, Anders Sterner

**Affiliations:** 1 University of Borås, Centre for Prehospital Research, Borås, Sweden; 2 Faculty of Caring Science, University of Borås, Work Life and Social Welfare, Borås, Sweden; 3 University of Borås, Academy for police work, Borås, Sweden; 4 Faculty of Health and Life Sciences, Linnaeus University, Växjö, Sweden

**Keywords:** Emergency Medical Services, Mobile Health Units, Interprofessional relations, Case study

## Abstract

**Introduction:**

Healthcare systems worldwide are facing numerous challenges, such as an aging population, reduced availability of hospital beds, staff reductions and closure of emergency departments (ED). These issues can exacerbate crowding and boarding problems in the ED, negatively impacting patient safety and the work environment. In Sweden a hybrid of prehospital and intrahospital emergency care has been established, referred to in this article as Medical Emergency Team (MET), to meet the increasing demand for emergency care. MET, consisting of physicians and nurses, moving emergency care from EDs to patients’ home. Physicians and nurses may encounter challenges in their healthcare work, such as limited resources for example medical equipment, sampling and examination, in unfamiliar varying home environments. There is a lack of knowledge about how these challenges can influence patient care. Therefore, the aim of this study was to explore the healthcare work of the METs when addressing patients’ emergency care needs in their homes, with a focus on the METs reasoning and actions.

**Methods:**

Using a qualitative multiple case study design, two METs in southwestern Sweden were explored. Data were collected from September 2023 – January 2024 and consist of field notes from participant observations, short interviews and written reflections. A qualitative manifest content analysis with an inductive approach was used as the analysis method.

**Result:**

The result of this study indicates that physicians and nurses face several challenges in their daily work, such as recurring interruptions, miscommunication and faltering teamwork. Some of these problems may arise because physicians and nurses are not accustomed to working together as a team in a different care context. These challenges can lead to stress, which ultimately can expose patients to unnecessary risks.

**Conclusion:**

When launching a new service like METs, which is a hybrid of prehospital and intrahospital emergency care, it is essential to plan and prepare thoroughly to effectively address the challenges and obstacles that may arise. One way to prepare is through team training. Team training can help reduce hierarchical structures by enabling physicians and nurses to feel that they can contribute, collaborate, and take responsibility, leading to a more dynamic and efficient work environment.

## Introduction

According to the World Health Organization (WHO), healthcare is facing several challenges, including an aging population [1] rising rates of chronic diseases, often characterized by exacerbation [2], which place greater demands on healthcare services. Simultaneously, the number of available hospital beds is decreasing, and due to staff cuts, there will be fewer ambulances and emergency departments (EDs) are closing [3]. In EDs, this leads to issues such as crowding and boarding, and which have a negative impact on the work environment such as workload that is too high, which may cause stress and risk of burnout [4]. Furthermore, crowding and boarding and have negative impacts on patient safety since of delays in medical treatment and inadequate monitoring, which can lead to increased mortality [5].

One way to meet patients’ needs for emergency care is to shift the care provided from the hospital to patients’ homes [6, 7]. Offering home-based care (HBC) has been shown to be cost-effective [8] and safe for patients [9]. However, it may entail longer treatment times than hospital care, especially for certain chronic conditions [10]. Studies indicate that exacerbations of chronic conditions such as heart failure and chronic obstructive pulmonary disease, as well as pneumonias [11], symptoms such as fever, dyspnea [12], nonspecific symptoms in frail elderly patients, patients with cognitive impairment [13], and pain or injury to the skeletal or muscular system [14] can be effectively managed at patients homes. Currently, there is no consensus on what HBC entails or how it can be termed [15]. Terms such as “Hospital At Home” [9, 16], “Same Day Emergency" [17], “Hospital In The Home” [18] or “Residential In Reach” [19] are used internationally, while in Sweden, general terms such as “Mobile teams”, “Mobile emergency teams”, or “Mobile home care teams” are used [20].

The Swedish healthcare system is divided into three levels of governance: state, region, and municipality. These levels are responsible for different parts of healthcare, specialized hospital care, primary care, and municipal care [21]. Currently, all levels are undergoing a transformation process called “good and integrative care” [22]. This initiative resembles the Integrated Care System in England [23] and aims to make healthcare more accessible and closer to the patient, focusing on their unique care needs [24, 25]. As part of the Swedish transformation process to meet the increased need for emergency care, a hybrid of pre- and intrahospital emergency care has been established [25]. This hybrid version of emergency care will, in this article be referred to as the Medical Emergency Team (MET). The MET, consisting of two organizations, ambulance services (AS), and EDs, has merged and operates outside the hospital setting. MET is not the same as care provided by ambulance, primary or municipal care, MET is rather a combination of these services. The MET is staffed with ED physicians and nurses from the ED or AS and provide emergency care to patients who have suffered from sudden illness or injury [26] and operates wholly or partially from hospital-affiliated EDs.

When emergency care is provided in patients’ homes, a holistic approach is required to ensure that all aspects of patients’ care needs, including medical, caring, physical, psychological, social, and existential needs, are addressed [27]. This means that METs must be prepared to handle a wide range of care-related issues with limited resources, in an unfamiliar environment to ensure that the care provided in patients’ homes meets their needs [28]. This requires the MET to collaborate across boundaries both within the MET, and outside the team with other care providers such as AS, primary care or municipal care [25, 29]. If the expectations of the MET’s care work, i.e., what they can do, are unclear, difficulties may arise [28]. In this study, healthcare work refers to performing various tasks which not only including technical skills such as collecting blood samples and managing medical equipment but also through understanding and responding to patients’ needs, both expressed and unexpressed. Furthermore, healthcare work includes communication within the MET, with patients and their relatives, as well as other healthcare actors. By examining how physicians and nurses reason and act when encountering patients’ care needs at home through the MET, obstacles and opportunities can be identified when hybrid emergency care is shifted to patients’ homes. The aim of this study was to explore the healthcare work of the METs when addressing patients’ emergency care needs in their homes, with a focus on the METs reasoning and actions.

## Method

Employing a qualitative multiple case study design [30], this study explored the MET as a contextually and socially bounded system [31]. The data were collected through participant observations, which enabled participation in daily activities, interactions, and events [32].

### Setting

The research settings were two METs in the southwestern part of Sweden: MET A, which operated from a hospital-affiliated ED, and MET B, which operated from the AS. The possible assignments providers for MET A and MET Bs were similar. However, MET B could have paced assignments identified by the ED and AS when the MET was not operational. MET B could also be assigned to time-critical medical conditions to make initial assessments/treatments while waiting for AS. Primarily, the nurses were responsible for checking the equipment and restocking supplies in the vehicle. When the MET had no assignments, the physicians in the MET A supported their colleagues in the ED, carried out administrative tasks, and answered incoming calls to the MET. The physicians in MET B had administrative tasks and handled incoming calls to the MET when the team had no assignments. The two METs had varying conditions and staffing, and the equipment was slightly different between MET A and MET B, consisting of up to 13 different units. For more information see Table 1.

**Table 1:** Setting for MET

	MET A	MET B
Geographical catchment area, population	6 municipalities 5. 142,1 sq. km 216 391 population	4 municipalities 1. 814,2 sq. km 107 692 population
Been in operation (months)	48	6
Operational hours	Physician: weekdays 7:30 AM-16 PM Nurse: weekdays 7:30 AM -16 PM and	Physician: weekdays 8:00 AM – 16 PM Nurse: weekdays 7:30 AM -17:30 PM
Type of vehicle	Passenger vehicle	Ambulance vehicle
Equipment	Respiratory care with oxygen. Equipment for measuring vital parameters such as blood pressure cuff, saturation meter, stethoscope, ear temp Patient monitor and defibrillator, Cor Pulse Materials for suturing and gluing wounds and different dressings. Material to be able to drain urine or insert an indwelling urinary catheter Diagnostic unit containing equipment for blood tests such as CRP, blood gas, white blood cell counts and urinalysis. Ultrasound Medicines to handle the most common emergency situations such as intravenous and oral antibiotics, pain reliever as Oxynorm, Oxycontin, NSAID. Warm intravenous fluids Aprons, mouth guards with and without filters, laundry bags for dirty material etc. Computer and associated printer. Folder containing medical records (WEST), information sheets for patients, relatives, and other caregivers as well as ID bands and death certificates.	Same as MET A Same as MET A Same as MET A Same as MET A Same as MET A Same as MET A, except for white blood cell count. Same as MET A but with fewer types and quantities of antibiotics, pain relivers and other medications Same as MET A Same as MET A Same as MET A
Additional equipment	Material to refill such as antibiotics and sampling equipment etc. Frenzel glasses	Maternity bag Cool box for transporting samples to the hospital.
No. Assignments/day for MET (range)	3 (1–11)	5 (1–10)
MET assignment description, limitation	Patients aged > 18 and frail elderly who suffer from treatable conditions that have occurred suddenly, not requiring advanced hospital-based resources.	Avoid emergency and inpatient stays for frail elderly, > 65 years and reduce ambulance assignments
Possibility for direct admission to ward	Yes	Yes
Documentations routines	Physician: Hospital record Nurse: Hospital record	Physician: Hospital record Nurse: Ambulance record
Possible assignment providers	Emergency dispatch Ambulance services Primary care facilities Municipality care Hospital outpatients	Emergency dispatch Ambulance services Primary care facilities Municipality care Emergency Departments follow-up Hospital at home team
Other		Possible to take on assignments concerning time-critical medical conditions to make initial assessment/treatment while waiting for ambulance resources.

### Study participants and recruitment

The study received ethical approval from the Swedish Ethical Review Authority in Stockholm (NO: 2023-02186-01) and access to the research field was granted and formally approved by the managers of the participating facilities. All physicians and nurses who staffed the MET were invited to participate in the study. MET A was informed by the first author through a staff meeting and email, while MET B received the information verbally from the medical chief of the department. Each participant received both oral and written information about the study from the first author and signed a consent form. Other ethical considerations regarding data protection and data security were followed in accordance with the Swedish Data Protection Act [33]. All data are presented at the group level for the purpose of ensuring and maintaining the participants’ integrity and confidentiality, and the study aligns with accepted ethical principles for research [34]. The studies included five physicians and five nurses from MET A and five physicians and five nurses from MET B, see Table 2 for further information.

**Table 2:** Staffing for MET

	MET A	MET B
Staffing	Physician (*n* = 6); Nurse (*n* = 6)	Physician (*n* = 5); Nurse (*n* = 5)
Gender	Male: 8 Female: 4	Male: 5 Female: 5
Age mean (years) Range (years)	45,8 30–61	42,1 34–59
Physicians work experience in hospital emergency care (years)	0–5 (*n* = 2) 6–10 (*n* = 2) 11–15 (*n* = 0) 16–20 (*n* = 2) > 21 (*n* = 0)	0–5 (*n* = 0) 6–10 (*n* = 2) 11–15 (*n* = 0) 16–20 (*n* = 2) > 21(*n* = 1)
Physicians work experience in MET (years)	< 1(*n* = 1) 1–2 (*n* = 5) 3–4 (*n* = 0)	< 1 (*n* = 4) 1–2 (*n* = 0) 3–4 (*n* = 1)
Education physicians	Medical practitioner (*n* = 2) Emergency medical specialist (*n* = 2) Senior Specialist (*n* = 2)	Medical practitioner (*n* = 0) Emergency medical specialist (*n* = 3) Senior Specialist (*n* = 2)
Nurses work experience in hospital and/or prehospital care (years)	0–5 (*n* = 0) 6–10 (*n* = 0) 11–15 (*n* = 0) 16–20 (*n* = 1) > 21 (*n* = 5)	0–5 (*n* = 0) 6–10 (*n* = 1) 11–15 (*n* = 1) 16–20 (*n* = 1) > 21 (*n* = 2)
Nurses work experience in MET (years)	< 1 (*n* = 0) 1–2 (*n* = 6)	< 1 (*n* = 5) 1–2 (*n* = 0)
Education nurses	Registered nurses (*n* = 0) Postgrad specialty in prehospital emergency care (*n* = 4) Postgrad specialty in primary care (*n* = 1)	Registered nurses (*n* = 1) Postgrad specialty in prehospital emergency care (*n* = 2) Postgrad specialty in anesthesia care (*n* = 2)

### Data collection

The data were collected during the period from September 2023 to January 2024 and consisted of participant observations with field notes [32], interview notes [35] and written reflections [32].

#### Observations

The first author conducted all observations by following both METs for full work-shifts, and each patient visit was defined as one observation. The duration of the observations varied between the METs, se Table 3. Physicians and nurses were encouraged to work as usual and to ignore the researcher, who aimed to maintain a low profile throughout. When arriving at the patient, the researcher was briefly introduced as a person who was there to observe how they worked.

**Table 3:** Overview of the data collection

	MET A	MET B
Observations		
Observation Days (*n* = 25)	12	13
Observed patient encounters (*n* = 73)	26	47
Observation hours	29	42
Observation minutes, mean (Range)	44 (14–104)	41 (2–82)
Driving time from start to arrival at address, minutes, mean (Range)	26 (2–61)	18 (1–51)
Time spent at the patient’s home, from arrival to the scene to door closed, minutes, mean (Range)	35 (10–57)	36 (2–71)
Field notes (number of words transcribed)	15 942	27 356
**Interviews**		
Mean (Range)	613 (240–1 400)	582 (253–1 018)
Interviews performed.	21	38
**Reflections** Reflections notes performed	23	47

All observations began when the MET received the assignment and ended when the door to the patients’ home closed. During the observation field notes were written containing what physicians and nurses said and how they reasoned when the assignment was received, during the assignment, and when it was completed. In total, 25 observation days were completed, comprising 73 observation instances. The observations lasted an average of 41 to 44 min and generated two to three pages of transcribed text, see Table 3 for further details.

#### Interviews

To obtain a deeper understanding of METs reasoning about their actions when patients’ care needs were met, the following questions were asked; What are your thoughts about the assignment and what are your thoughts on the teamwork? Follow-up questions were posed in response to the answers given. To gain a deeper understanding, questions such as “Can you tell me more?” were used frequently. The interviews took place after the observations were completed, conducted in the car while leaving the patient.

#### Reflection

After the completion of the observation and interviews, the first author wrote down reflections in a reflective text. The purpose of the reflection was to gain additional understanding of the research questions. These reflections were utilized in the discussion of the results.

### Data Analysis

The collected data consisted of field notes, interview texts, and reflection texts were transcribed by the first author. During transcription, the text became more descriptive than the original because several fieldnotes were written with incomplete sentences when trying to write down as much as possible. The data were sorted into three phases of the MET assignment- preparatory, during the patient visit, and the reflection phase - which is a way to structure the data chronologically and provide organization [30]. To ensure that the analysis was as free as possible from interpretations, the author group discussed och reflected during the process. The qualitative manifest content analysis was conducted using an inductive apporach [36] and began with the first author reading the fieldnotes and interview texts multiple times to understand the content and obtain an overall sense of the data. In the second step, units from the text were extracted that addressed the aim of the study, to capture and describe METs healthcare work such as communication, physical actions, understanding and responding to patients’ care needs. These units were condensed without losing the content and coded based on their content. The codes were then sorted into categories and subcategories describing different aspects, similarities, or differences, ultimately forming four categories: Assignment reception and preparation phase, patient interaction and examination phase, decision-making and treatment phase and reflection and evaluation phase.

## Results

The results will be presented in chronological order, from when the METs receive the assignment until the assignment is completed, concluding with reflections from the METs. The results will include situational descriptions and quotes to present general patterns for MET A and MET B; unless otherwise specified, the aspects were the same. Each phase begins with a generic vignette that encompasses of several observation sessions. Individual observations are presented with the unique observations number.

### Assignment reception and preparation phase

*The METs are on their way to a patient*,* the physician reads loud from the patient’s medical record*,* the phone rings repeatedly*,* regarding new assignments and questions from AS*, municipality care, *etc. After each call*,* the physician gives a summary to the nurse. The nurse asks “inquisitively”… which patient are you referring to? The one we’re heading to*,* or is it another? Transportation time is then spent with the physician dictating notes in the patient’s medical records where recommendations to seek other levels of care or stay at home are given. When the nurse parks the vehicle outside the patient’s address*,* the METs discuss which equipment to bring.*

Patient assignments could be provided at any time during the shift via phone or radio, and the information was sometimes vague or incomplete. The time for preparation varied depending on when the assignment was received, where the METs were geographically located in relation to the patient’s address, whether in an apartment in the same building or several kilometers away. Physicians received the most calls; occasionally, the speakerphone function was used so that the nurse could take part in the conversation and ask questions. On occasions when nurses answered the phone, a brief report was taken, and the nurse was asked to call back after consulting with the physician, or the phone was handed directly to the physician. Unlike MET A, MET B could receive assignments from the ED and AS when the MET was not operational. Messages were then written on notes handed over in person during shift changes at the ambulance station or at the ED. MET B could also be assigned to a critically ill patient, resulting in all delays for all other accepted assignments. On some days, assignments could pile up, causing patients to wait for several hours or for the METs to decline assignments. When assignments were received, the METs discussed the pros and cons to determine if it was a suitable patient; the physician had the authority to accept the assignment.

Nurses drove the vehicle, and transportation time could occur in silence, with the phone ringing incessantly, or with the METs discussing private matters. Physicians read and documented in the patient’s journal for upcoming and completed patient assignments. The METs could have difficulties finding the correct address; the functionality of the navigation system varied, and on several occasions, it did not work at all or provide incorrect directions. Upon arrival at the correct address, the need for additional information, such as a gate code or miscommunication regarding contagious patients, was discovered. When the vehicle was parked outside the patient’s residence, the decision on which equipment to use was made. Physicians were responsible for bringing the laptop bag and ultrasound equipment, while nurses were responsible for carrying other equipment. In instances where physicians were in an ongoing call, the nurse entered the patient’s home alone, but usually, the METs entered together.

### Patient interaction and examination phase

*When the METs entered the patient’s home*,* the physician approached first*,* either standing or squatting in front of the patient and said: Hello*,* my name is xxx and I am a physician*,* how are you? The nurse stands quietly behind*,* not wanting to interrupt the patient’s conversation with the physician and beginning to retrieve and set up the lab equipment. The physician examines the patient*,* is interrupted several times by phone calls*,* and then prescribes which tests to take. The nurse*,* who has been in another room*,* is not prepared for which tests to take and does not understand why.*

When the METs arrived at the patient, they introduced themselves by name and title, and that they were from the MET. The physician was often the first to reach the patient. In instances where the MET had been assigned a critically ill patient, which was a part of MET B’s mission, there were usually already one or two ambulances on site. The physician then first contacted the ambulance nurse. When MET B was the first unit on site, the physician took the medication unit and went in alone to see the patient while the nurse parked the vehicle and brought in the rest of the equipment.

After the introduction, physicians usually immediately began gathering information about what had happened and how the patient was feeling. This meant that the nurse did not have a natural opportunity to greet, which could result in the nurse’s introduction occurring later during the visit or being completely omitted. Physicians often choose to sit down beside the patient or squat down. Before the examination, lights were sometimes turned on, blinds were pulled up, and the bed was raised. This was sometimes initiated by the patient, other individuals present, or the METs themselves. Examinations could also be conducted by leaning over the patient, in dim light where mobile flashlights were used to read vital signs. Depending on how many other people were in the room, information about the sequence of events could come from multiple sources. Nurses sometimes chose to listen as physicians gathered information, sometimes asked questions, or assisted when communication between the patient and physician did not work. When several people were present, it could sometimes become noisy in the room, resulting in the patient not hearing or understanding what the physician was asking, and the patient’s voice not being heard. The METs could be interrupted several times by phone calls with requests for new assignments, pending assignments, and advisory calls from AS.

The examinations were conducted based on the ABCDE principle (airway, breathing, circulation, disability, and exposure) and were carried out by a physician, while the nurse performed the examination, when agreed upon during the preparation phase. Physicians always listen to patients’ lungs. The nurse sometimes participated in the initial examination by handing equipment such as a stethoscope to the physician or standing quietly by the side and listening. Unlike in the MET A group physicians in the MET B group were interested in improving nurses’ examination techniques, such as listening to the lungs and interpreting electrocardiograms (ECG). Physicians encouraged the nurses to listen and report what they heard or allowed the nurses to make the initial assessment of the ECG. Different examination findings were discussed openly, which could lead to various expressions of curiosity and questions among those present. Most often, the nurse chose to begin measuring vital parameters (respiratory rate, saturation, blood pressure, pulse, and temperature) or to prepare the laboratory equipment during the ongoing examination. The cold blood pressure cuff was warmed on rare occasions. The clothing of patients could be partially or fully removed during the examination and was not routinely returned after the examination. Once vital parameters had already been taken, the nurse waited to take new until the physician indicated a desire for them. Nurses could express concerns about patients’ well-being, such as affected vital parameters during the ongoing examination, which the physician did not confirm or did not consider noteworthy.

*The nurse measures the patient’s saturation… looks at the meter… furrows brow in concern*,* asks the patient to take a few deep breaths. Says to the physician: …are you noting the value? Yes*,* says the physician*,* who continues to sanitize the equipment* [Observation 45].

Problems that could arise when vital parameters were taken included that they were often said out loudly in the room, which colleagues did not always hear. The values could be noted on journal sheets, pieces of paper, on gloves, or not at all. This resulted in uncertainties about which parameters had been taken and what they showed. The mission of MET B, unlike that of MET As, was to care for elderly patients. They could be interrupted during ongoing examinations to care for another patient, residing in the same assisted living facility, who had suddenly deteriorated. In those instances, the nurse stayed with the patient and continued the examination.

Sampling, which occurred after a physician’s order, was performed by nurses. Sometimes, the nurse could interrupt the ongoing examination to obtain blood samples without a physician’s order; other times, the nurse stood by and waited, ready with the sampling materials. When the nurse took the samples, the physician usually chose to sit down in another room to read the patient’s journal and plan for potential treatment. Nurses were responsible for retrieving the laboratory equipment and placing it where there was sufficient space, usually in an adjacent room; patients were then left alone while blood samples were analyzed. The results from sampling were crucial in some cases, such as when patients could not participate in the visit due to a disability. Blood samples could be taken via arterial, venous, or capillary methods, with the choice of method varying. In MET A, it was the patient’s symptoms and signs determine the choice of sampling method, while in MET B, arterial or capillary blood samples are usually taken. The reason for choosing the sampling technique was unfamiliarity with the venous sampling technique and the nurses’ interest in learning to collect arterial samples. This resulted in patients being punctured multiple times, and the decision regarding sampling could suddenly be re-evaluated when the sampling failed when there was a lack of available analysis material.

The issues that could arise with laboratory equipment included its sensitivity to cold temperatures and the shortage of the special cards. Attempts to warm the laboratory equipment were made by placing it near warm sources in the patient’s home, warming it against the body, and re-evaluating the need for sampling. MET A chose to place the sensitive equipment in another location in the vehicle, which MET B did not have the opportunity to do. The lab equipment was space-consuming, which challenged the METs in homes with many personal belongings and dirty surfaces. MET A, which had more lab equipment than MET B, forgot part of the equipment at the patients’ homes. METs can carry up to seven units into the patient, depending on the patient’s condition. Space constraints combined with large jackets during cold weather caused patients personal belongings to fall to the floor and break.

### Decision-making and treatment phase

*Physicians made decisions regarding treatment*,* which could involve medication*,* palliative care orders*,* expanded sampling*,* and continued hospital care. Physicians discussed treatment options with patients*,* when possible*,* as well as other healthcare personnel present. The nurse*,* who often remained in another room to manage patient sampling and pack equipment*,* did not hear the discussions and thus was unprepared for potential treatment and lacked knowledge of prescribed medications. When the decision for hospital transport was made*,* the nurse arranged it while the physician documented.*

Decisions about treatment were typically made by physicians during the examination or sampling phase and could involve medication, expanded sampling, continued hospital care, palliative care orders, or observation. During this phase, METs could be interrupted repeatedly, resulting in incomplete perceptions of orders and important decisions made. Nurses repeated the current medication orders and awaited confirmation from the physician before administering the medication. Nurses could ask patients and relatives several questions but did not wait for or expect a response. Physicians usually provide medical self-care advice, while nurses ask if they have sufficient support from other healthcare providers. Nurses could also take initiative and suggest treatments to patients who had not communicated with the physician, which could sometimes lead to misunderstandings regarding patients’ degree of illness.

*Patient with diarrhea*,* vomiting*,* high fever*,* and dizziness for five days. The patient said*,* “I find it hard to drink”. Nurses responded “ … it is a shame to go to the hospital*,* better to stay at home. You should take paracetamol and ibuprofen regularly throughout the day for the fever*,* and then you must drink properly*,* preferably soup or oral rehydration solution”. Meanwhile*,* the physician stands a short distance away*,* looks worried*,* makes a few attempts to intervene in the conversation but fails and eventually gives up* [Observation 45].

Physicians typically proposed treatment options to patients, and in cases where patients had conditions such as impaired cognitive abilities or were in the end-of-life stage, they were not involved in decision-making. Decisions were discussed with other healthcare personnel if they were present. Relatives were involved when possible, and some decisions required physicians to try to help the patient understand, such as patients with mental health issues. The mission of MET B included, for example patients who experienced cardiac arrest and patients who died. During these missions, the MET took the time to talk about and support the relatives present and reassured them that the patient had not suffered.

The familiarity with handling the medications that METs carry varies. Nurses in the MET A were accustomed to administering the medications typically used in the ED, such as antibiotics, unlike those in the MET B. When questions and uncertainties arose regarding medications, which could concern how antibiotics should be diluted and administered, nurses consulted physicians, but they lacked practical knowledge. They then searched for information together on the internet or called the hospital’s ED for advice. On occasion, prescribed medication was not given because both the physician and the nurse lacked knowledge of how the medication should be administered.

*Patient with suspected sepsis… the MET has called an ambulance… Physicians have ordered intravenous antibiotics. The nurse asks;… should we skip giving the antibiotics… the ambulance will be here soon?* [Observation 42].

When multiple tasks needed to be performed, physicians could offer to administer medications. Since physicians were not familiar with the units containing medications and equipment, nurses had to interrupt their ongoing tasks to show the physician which unit the equipment was in and how it worked.

Patients who expressed insecurity about staying at home or being too ill were offered hospital care. The nurse arranged transportation to the hospital, assisted in moving the patient from, for example, the bed to the ambulance stretcher, and was responsible for filling out the journal sheet accompanying the patient to the hospital. The physician was responsible for documentation and contact with the receiving unit. When the physician had a probable working diagnosis and when there were available beds in the hospital wards, patients could be admitted directly. However, when there was a shortage of beds, which was common in MET A, or when the diagnosis was unclear, patients were transported to the hospital’s emergency department for further evaluation, treatment, and waiting for an available bed. The physicians were always documented in patients’ journals, while the extent of nurses’ routine documentation varied. The differences included nurses in the MET A documenting the reason for the visit, nursing status, entering test results, updating interventions from community care, and phone numbers for the patient and relatives in the patient’s hospital journal. MET B’s nurses documented by creating a case log in an ambulance journal, with reference to the physician’s notes in patients’ hospital journal.

The other healthcare providers with whom the METs collaborated with varied depending on the differences in the mission descriptions. Cooperation with municipal care was common, and physicians were responsible for handovers. MET visits often include takeovers, which could consist of newly prescribed medications, administration of antibiotics and intravenous fluids, as well as vital sign monitoring. There were regulations at certain special accommodations in MET B’s catchment area that governed, for example, the use of IV stands inside patients’ rooms. This resulted in the application rule being broken at the MET initiative when a patient needed intravenous fluids. The extent to which the prescribed medications were left varied. MET A left newly prescribed medications, either for the entire treatment period, which last up to 10 days, or for the first two to three days. Intravenous antibiotics were always left for the first day, then a follow-up visit was usually scheduled for the next day, or the patient could transition to oral treatment. In MET B, the first dose of antibiotics was given intravenously, and possibly the first tablet dose, with the remaining doses prescribed by the physician.

When the mission was considered completed, it was usually the nurse who sanitized the equipment and packed it. The MET usually said goodbye together and tried to restore the patient’s home to how it was when they arrived. Nevertheless, on occasion bright lights were forgotten to be turned off, the patient’s bed was not turned down, and that the patient would not become cold was not ensured. Usually, the nurses carried the equipment to the car, while the physician was responsible for the computer and printer and possibly the ultrasound on occasion.

### Reflection and evaluation phase

The METs reflected on whether the mission had involved an ‘appropriate patient’ and considered whether additional examinations that the METs did not perform, such as X-rays, could have affected or improved the quality of care. Patient benefit was viewed as crucial, where the METs considered patients’ preferences alongside potential risks of staying at home, such as an increased risk of falling. The assigned missions often concerned patients who could be effectively treated at home, where a visit to the ED would not have added value.

*The assignment involved a patient with addiction problems. The apartment was filled with cigarette smoke*,* with stacks of newspapers along the walls and personal belongings scattered everywhere. The MET had been contacted by home healthcare. The patient was not very responsive during the examination* [Observation 9]. *The doctor said; “The patient would have been sent to the ED if the MET had not assessed and treated the patient at home. However*,* an ED visit would not have made any difference to the patient’s outcome”. The nurse added: “I noticed he was so tired and lethargic… he seemed affected.” The physician responded*,* “…I had no thoughts of that at all”* [Interview 9].

However, the METs also acknowledged that some missions required skills they did not possess, particularly in psychiatry. They expressed uncertainty about their role in certain missions and believed some were better suited for ambulance care, such as patients needing oxygen therapy. For patients requiring oxygen, the METs felt hospital care was necessary and that their involvement could delay treatment. Missions solely based on telephone assessments of patients’ needs were often considered less reliable compared to those assessed by licensed personnel on site. Patients’ emergency care needs varied, from requiring rapid hospital transport to care within primary care settings. The METs noted that some missions were not about providing home care but rather about optimizing ambulance resources, using methods like stretcher transport or a single-nurse ambulance. The METs agreed that in some cases, patients had waited too long for an ambulance and needed quicker intervention.

The METs expressed that within the team, there was an enabling and safe climate where they complemented each other and worked beyond professional boundaries, which they considered a strength. However, nurses sometimes felt that their skills were underutilized in missions that solely involved transporting physicians to patients. Nurses in the MET B group perceived ambiguity in their professional roles, while those in the MET A group experienced inequalities in task distribution. They expressed feeling responsible for multiple tasks, which could be time-consuming and challenging, such as checking vital signs, conducting tests, and addressing patients’ care needs, where they believed physicians could offer more support. The METs highlighted several strengths in teamwork, such as having one team member communicate with the patient to establish a strong connection and contribute different perspectives, with doctors focusing on the medical aspect and nurses on the care perspective. While the METs felt confident in the medical aspect, physicians found nursing tasks challenging, including assessing patients’ nutrition, elimination, personal hygiene, and fall risk assessment.

*The mission involves an elderly patient in a nursing home with deteriorated general condition*,* diagnosed with dehydration by the time the MET leaves the patient* [Observation 14]. *On the way back*,* the nurse says; ”The patient resides in a facility*,* and it is not our responsibility to take over the facility’s duties. Since the patient did not express a desire for anything to drink*,* nursing interventions can be deprioritized in favor of other patients who are waiting* [Interview 14].

The METs reflected on whether the decisions made were right or if they could have done things differently. Physicians in MET B viewed receiving many questions as positive because it prompted deeper thought. There was a clear need for confirmation among physicians during missions involving difficult-to-assess patients or making challenging decisions, such as end-of-life discussions and initiating palliative care orders. However, this need for confirmation was not always recognized by colleagues. Instead, nurses expressed concern about the lack of written information detailing the actions taken and the treatment plan implemented.

## Discussion

The results of the multiple case study indicate that physicians and nurses face several challenges in their daily work such as recurring interruptions, miscommunication and faltering teamwork. This can lead to stress, which not only exposes patients to unnecessary risks but also negatively affects physicians and nurses [37]. One way to attempt to understand and interpret the work systems within which physicians and nurses operate within is to investigate what happens within and outside the MET and how it can affect caregiving [29].

The results indicate that the MET could be interrupted multiple times during a patient visit by incoming calls regarding potential new patient assignments, ongoing consultations, or advisory calls from, for example, the AS. Additionally, as described in the *assignment reception and preparation phase*, MET B could be assigned to a critically ill patient. These interruptions could cause ongoing examinations to be disrupted and force physicians to start over, resulting in inefficient work. Constant interruptions can create feelings of losing control, leading to dissatisfaction and stress, which can result in burnout over time [38]. Emergency physicians and nurses are more frequently affected by burnout and emotional exhaustion [39]. Interruptions can negatively impact their ability to concentrate, potentially leading to inadequate or incorrect decisions regarding the care and treatment required for the patient’s condition [40]. In addition, the MET did not have necessary information such as access codes, and lacked knowledge about whether patients were carrying infectious diseases such as COVID-19 or gastroenteritis. Sometimes, the physician had received this information but had not shared it with the team. The failure to have such information exposed the MET to unnecessary risks of either contracting infections themselves or spreading them further. Previous research indicates, for example, that staff in AS are at greater risk of acquiring infections due to the uncontrolled environment in which they work [41].

Physicians were often the first to acknowledge the patient and would begin taking the medical history when MET arrived unless it involved a critically ill patient, which could be the case in MET B. On those occasions, as described in *the patient interaction and examination phase*, the physician took on a more withdrawn role. It was evident during the observations that the AS were accustomed to handling these situations and that the METs medical contribution was limited. Many patients who received care and treatment from the MET, especially MET B were elderly residents living in nursing homes. On several occasions, the MET expressed that these elderly patients were ideal candidates for emergency care at home, but also perceived that many of the visits would have been more appropriately managed by primary care. This is supported by previous research, which shows that emergency physicians and nurses perceived a lack of competence and insufficient involvement in patient care as contributing factors to AS being called out and the patient being transported to the ED [42].

During the examination, physicians might ask the nurse to measure vital signs, hand over a stethoscope, or remove the patient’s clothing to facilitate a more thorough examination This approach could be due by the fact that physicians working in EDs are accustomed to having limited time for gathering necessary information for making treatment and diagnosis decisions [43]. Medical history and examination results sometimes occurred simultaneously but could also occur separately. The questions asked were often open-ended, such as ”How are you feeling?” and ”Can you tell me why we are here today?”. Nurses often choose not to participate during the physician’s examination, as *described in the patient interaction and examination phase*. Instead, they prepared the lab equipment and carried out the physicians’ orders, acting as assistants. MET A, had more lab equipment to prepare than MET B, which could be time-consuming to unpack and set up. This withdrawn role that nurses sometimes adopted could lead to care becoming primarily medically focused, potentially overlooking patients’ comprehensive care needs. It is not surprising and not a new phenomenon that emergency care primarily has a medical focus [44]. Previous research shows that in EDs, there are deficiencies in both identifying and responding to patients’ fundamental care needs, such as nutrition, elimination, and fall prevention, which can lead to adverse events [45]. MET A was more likely to follow the ED’s routines and guidelines, such as documenting provided care and collecting blood cultures before administering intravenous antibiotics—a practice that was not followed at all in MET B. By adhering to these guidelines, MET A not only ensured compliance with established protocols but also enhanced patient safety. Guidelines are an essential tool for providing updated information and increasing the standard of [46]. In conclusion, while the medical focus in emergency care is undeniably important, integrating a comprehensive approach that includes adherence to guidelines is crucial, especially since this type of mobile care is primarily provided for frail elderly patients [25].

One way to increase patient safety and quality of care could be to work in teams [47] where collaboration is highly emphasized [25]. Collaborating is important in all care context, but is especially crucial in emergency care, where decision need to be made rapidly with limited information [48]. When emergency care is delivered in patients’ homes, MET face several challenges, including weighing the benefits and risks of providing care at home while also considering the patient’s wishes and autonomy [49]. The results of this multiple case study indicate that teamwork in the MET could be insufficient. Physicians and nurses had differing perceptions of the goal of the patient visit. A possible explanation for this could be a lack of sufficient communication between physicians and nurses. Nurses were not always involved when assignments were accepted, resulting in them having little or inadequate information when they arrived at the patients’ homes. During patient visits, physicians and nurses often worked separately, indicating a sequential working method, as described in *the patient interaction and examination phase*. A work system consists of several interdependent parts with various characteristics that rely on each other, making caregiving complex [29]. A sequential working method can thus contribute to unsynchronized, inefficient care, with risks for patient harm, such as missed nursing interventions or the failure to treat time-critical conditions according to standard protocols, such as early administration of antibiotics in suspected sepsis patients [50]. Another possible explanation for physicians and nurses working separately could be hierarchical structures within the MET. These hierarchical structures might have included ambiguities regarding professional roles and who was expected to be responsible for and carry out different parts of the healthcare work when identifying and meeting patients’ care needs [50]. In addition, previous research has highlighted the necessity of shared responsibility for patient care, which develops over time [51]. Another explanation could be that both METs were relatively new, involving a completely new way of working for which physicians’ and nurses were not trained for.

However, this study reveals that the phases described in the results can happen at any time and affect each other, underscoring the complexity the MET encounters when managing patients’ care at home. These factors, when combined, can negatively impact both care and patient safety [50] especially if the skills within the MET are not fully utilized. To address this, it is suggested that interprofessional simulation be implemented. This approach brings together different disciplines, allowing them to practice collaborative care in a controlled setting, which could enhance patient safety [52].

### Strengths and limitations

A strength of this study is that it was conducted as a multiple case study, which is more compelling and robust than single case study [30]. Data also describe current phenomena in their real-world context, which is advantageous when the boundaries between the phenomenon and the context are unclear [30, 32]. Another strength is that several approaches were used to gather data such as participant observations, short interviews and reflections. This enabled triangulation, which is a method used to explore complex phenomena that cannot be fully understood, with a single method or data source [53], can provide a broader and deeper understanding of physicians’ and nurses’ healthcare work in this hybrid form.

However, there are also some limitations to acknowledge. When the study was conducted, the METs were relatively new, which may have led to certain issues related to their ongoing development. To gain access to the research environments, gatekeepers were used. This can be seen as a weakness since gatekeepers are often key individuals within the organization with certain power, which may have influenced the participants to take part in the study to appease the chief. There were also differences in the number of observations between the METs. A reason for this was METs differed in missions and geographic catchment areas. The size of the area they served may have affected the number of completed observations due to the time they spent traveling between patients’ homes. Technical differences regarding the vehicles between the METs, as well as the inability to control incoming phone calls, may have resulted in important information being overlooked.

Finally, a limitation may be the professional role of the observer as a licensed nurse, which complicated maintaining the researcher role. On a few occasions, the first author had to abandon the observer role to assist with equipment and medication, which may have led to some data not being recorded. However, patient safety was a priority.

## Conclusion & implications

This study highlighted the challenges physicians and nurses meet when a new service is launched in emergency care. The challenges include the expectation for physicians and nurses to collaborate in teams, ambiguity in job descriptions leads to inefficiencies and uncertainty. Moreover, physicians and nurses are not accustomed to working together, and team compositions change almost every shift. As a result, established work routines are difficult to maintain, requiring team members to constantly adapt to new colleagues and workflows.

It is also important to note that these challenges can contribute to increased stress levels among staff, which can negatively impact patient care. When there are deficiencies in communication and collaboration within the team, this can lead to mistakes or delays in care, exposing patients to unnecessary risks. To counteract these problems, it is crucial to invest in team training and to develop clear job descriptions and routines that support effective and coordinated teamwork. Team training can help reduce hierarchical structures by enabling physicians and nurses to feel that they can contribute, collaborate, and take responsibility, leading to a more dynamic and efficient work environment. By practicing reflection and feedback after completing assignments, a more inclusive and development-oriented environment can be fostered, which in turn can positively impact the care provided by METs.

In summary, the study shows that it is essential to place great emphasis on planning and preparation when introducing new forms of care such as MET. By ensuring that all team members are well-prepared and that there are clear structures and support in place, a more dynamic and efficient work environment that benefits both staff and patients can be created. This hybrid version of prehospital and intrahospital emergency care is a complement to traditional hospital care, ED, AS, primary and municipal care. This requires collaboration between different organizations and staff categories, where patients’ current needs and situations are the focus, without boundaries. Further research is needed to define or explain what MET entails or how it can be termed. Likewise, can physicians and nurses experience to meet patients emergency care needs at their homes provide valuable insights.

## Data Availability

The datasets used and/or analyzed during the current study are available from the corresponding author on reasonable request.
